# The feasibility of generalized face mask usage during the COVID-19 pandemic: a perspective from Latin America

**DOI:** 10.1017/ice.2020.227

**Published:** 2020-05-11

**Authors:** Daniel Moreno Soto, Walter D. Cardona Maya, Esteban Londoño Agudelo, Julio C. Bueno-Sánchez

**Affiliations:** 1 Reproduction Group, Medical School, University of Antioquia, Medellín, Colombia; 2 Faculty of Medicine, CES University, Calle 10A #22-04, Medellín, Colombia; 3 Institute of Tropical Medicine, Antwerp B-2000, Belgium

*To the Editor—*Standardized medical masks, such as surgical or procedure masks and face-piece respirators, including N95 and N99, are an indispensable part of the protective personal equipment (PPE) during infectious outbreaks. These masks are intended for single use by healthcare personnel during high-risk procedures.^1,2^ However, the reuse of masks, as well as the extension of the usage to the general population, commonly arise during a crisis like the current COVID-19 pandemic. Official recommendations about this generalized usage have varied during the outbreak and according to the available evidence. The World Health Organization currently recommends restricting medical masks to healthcare workers and people with respiratory symptoms or caring for a person suspected of SARS-CoV-2 infection, along with diligent compliance to other infection prevention and control measures, such as hand hygiene and social distancing.^1^ However, new evidence seems to indicate that the use of facial protection by the general population may help to prevent the overall transmission of SARS-CoV-2.^3^

As the pandemic advances in the Americas, with 1,857,509 confirmed cases and 111,942 deaths as of May 15, 2020,^4^ some Latin American countries, such as Colombia,^5^ have established measures forcing the general population to wear facial protection in public settings. The extent of the benefit of such measures is still hard to assess, but as a precautionary principle, it could be preferable to use any kind of mask in case it entails some improvement. However, such a premise would only hold if there is a fair trust that this setting will most likely not generate additional risks during the outbreak. Briefly, the measure must be applied under some parameters to be safe.

First, mass use of facial protection requires that a sufficient and sustained supply of medical masks for healthcare staff is available.

Second, in the recurring scenario of insufficient supply due to shortage, the necessity of disinfection processes for lengthy usage or reuse of face masks must be acknowledged. The careless extension of intended operation limits could lead to self-contamination. Recent evidence shows that the virus might be particularly stable on the surface of surgical masks (one of the currently most widely used face mask types) for as long as 7 days.^6^ There are several feasible alternatives for disinfecting face masks, with some type of heat and ultraviolet germicidal irradiation standing out among them. These methods have been shown to successfully inactivate the virus^6-8^ without causing significant loss of mask functionality,^9^ and they are already being used in healthcare facilities, either by using autoclaves or by repurposing incubators, idle cabins, or whole rooms with ultraviolet type C (UVC) light bulbs.

Third, the accessibility of the general population to mask disinfection methods must be assessed. Chemical methods of disinfection, such as soap, ethanol, or sodium hypochlorite, are not viable because they would remove the electrostatic charge of the mask, impairing its filtration capacities.^10^ An alternative for mask disinfection is devices crafted to work with UVC or heat, and for which building instructions using commercially available materials can be found online for free. However, a large portion of the population will not have access to these devices; therefore, the use of washable cloth face covering should be favored; it is the current trend in some countries.

Fourth, independent of the type of masks used, people must be systematically educated about its proper use and actual capabilities (eg, to change washable face masks every 3 hours with a clean one and maintain social distancing).

Overall, the application of mass masking has an intrinsic risk that must be evaluated. Latin American countries are particularly vulnerable because they are in general not self-sufficient to ensure an adequate procurement of masks during the outbreak, which will pose a threat to the safety of already vulnerable healthcare personnel, and the low income of the population will probably result in excessive reuse of masks. Moreover, this region has no prior experience with mask use, unlike many Asian countries, such as China, South Korea, or Singapore, which are usually the major reference for masking measures. Ultimately, sociopolitical constraints may prompt a premature end to social distancing, and negligent face mask usage could induce a false sense of security based on insufficient information.
